# Gestational diabetes mellitus and the role of intercurrent type 2 diabetes on long-term risk of cardiovascular events

**DOI:** 10.1038/s41598-021-99993-4

**Published:** 2021-10-27

**Authors:** Jiyu Sun, Gyu Ri Kim, Su Jin Lee, Hyeon Chang Kim

**Affiliations:** 1 Medical Research Collaborating Center, SMG-SNU Boramae Medical Center, Seoul, Korea; 2 Department of Preventive Medicine, Yonsei University College of Medicine, Seoul, Korea; 3 Institute of Health Services Research, Yonsei University, Seoul, Korea; 4 Department of Internal Medicine, Seoul Red Cross Hospital, Seoul, Korea

**Keywords:** Diseases, Endocrinology, Risk factors

## Abstract

Recent studies have shown that gestational diabetes mellitus (GDM) is associated with an increased risk for cardiovascular disease. GDM has also been shown to be a risk factor for type 2 diabetes (T2DM) after pregnancy. However, there is limited evidence regarding the role of intercurrent T2DM on the relationship between GDM and future CVD. Thus, we investigated the risks of incident cardiovascular events among women with GDM during pregnancy compared to women without GDM and whether the increased CVD risk is dependent on intercurrent development of T2DM. We conducted a population-based retrospective cohort study using the Korean National Health Insurance Service claims database. Outcomes were the first occurrence of any CVD (myocardial infarction, treatment with coronary revascularization, heart failure, and cerebrovascular disease). Cox proportional hazard models were used to assess the association between GDM and incident CVD events, using landmark analysis at 4 years. A total of 1,500,168 parous women were included in the analysis, of which 159,066 (10.60%) had GDM. At a median follow-up of 12.8 years, 13,222 incident cases of total CVD were observed. Multivariable-adjusted hazard ratio for total CVD among women with prior GDM, compared with those without GDM, was 1.08 (95% CI 1.02–1.14). Further classifying GDM by progression to T2DM in relation to total CVD risk indicated a positive association for GDM with progression to T2DM vs no GDM or T2DM (HR 1.74; 95% CI 1.40–2.15), and no statistically significant association for GDM only (HR 1.06; 95% CI 1.00–1.12). GDM with subsequent progression to T2DM were linked with an increased risk of cardiovascular diseases. These findings highlight the need for more vigilant postpartum screening for diabetes and the implementation of diabetes interventions in women with a history of GDM to reduce future CVD risk.

## Introduction

Epidemiological studies have established that gestational diabetes mellitus (GDM), characterized by transitory form of diabetes induced by glucose intolerance and pancreatic β-cell dysfunction with onset or first recognition during pregnancy, is associated with cardiovascular risk factors such as type 2 diabetes (T2DM)^1–3^. In a meta-analysis of 20 studies with more than 670,000 participants, GDM has been shown to confer more than sevenfold increased risk of developing T2DM^4^. However, there is limited and inconclusive evidence for the effects of GDM on long-term risk of cardiovascular disease (CVD) and it is uncertain whether such association is dependent upon intercurrent progression to T2DM^5–8^. National health registry data from Sweden, showed that women with GDM have a considerable risk of developing CVD, even after adjustment for subsequent T2DM^3^. Yet, other studies have found that controlling for T2DM fully attenuates the increased risk of CVD in women with diagnosis of GDM in pregnancy^6,7^.

With the absolute number of deaths attributable to CVD rising steadily from 12.31 million in 1990 reaching 18.6 million in the year 2019, CVD is the leading global cause of mortality and disability-adjusted life years^9^. Although the CVD mortality rates are much lower among women compared with men, CVDs, principally coronary heart disease and stroke, are responsible for over 8.6 million deaths among women per year^10,11^. Given the increasing global burden of cardiovascular diseases among women, it is of great public health significance to identify factors that contribute to worsening CVD risk profiles. Accordingly, we sought to investigate the relationship between GDM and the risk of cardiovascular events using the Korean National Health Insurance Service (NHIS) database and examine the role of intercurrent T2DM in determining CVD outcomes in women with previous GDM.

## Results

### Participant characteristics

1,500,168 parous women aged 20–49 years were available for analysis, of which 159,066 (10.6%) reported having GDM while 1,341,102 (89.4%) women did not have GDM. General characteristics of the study population at index date according to GDM status are summarized in Table 1. Compared with non-GDM women, those with GDM were generally more likely to be older and were more often in the higher income groups. Women with GDM were also more likely to have a history of preeclampsia or hypertension, polycystic ovary syndrome, as well as history of dyslipidemia than individuals without GDM. However, no statistically significant difference in parity number was detectable between the two groups.

**Table 1:** General characteristics of the study population at index date according to gestational diabetes mellitus diagnosis.

	GDM diagnosis						P-value^†^
	Total		No		Yes		
	N	%	N	%	N	%	
Number	1,500,168	100.00	1,341,102	89.40	159,066	10.60	
**Age at index date, years**							< 0.0001
20–24	119,566	7.97	111,559	8.32	8007	5.03	
25–29	624,771	41.65	560,366	41.78	64,405	40.49	
30–34	589,440	39.29	523,347	39.02	66,093	41.55	
35–39	149,382	9.96	131,082	9.77	18,300	11.51	
≥ 40	17,009	1.13	14,748	1.11	2261	1.42	
**Socioeconomic status**							< 0.0001
Low income	335,050	22.84	299,590	22.86	35,460	22.73	
Middle-low	423,376	28.86	379,103	28.92	44,273	28.38	
Middle-high	471,121	32.13	420,816	32.10	50,305	32.25	
High income	237,242	16.17	211,291	16.12	25,951	16.64	
**Total parity**							0.2524
1	1,283,451	85.55	1,147,395	85.56	136,056	85.53	
2	214,411	14.29	191,670	14.29	22,741	14.30	
≥ 3	2,306	0.15	2,037	0.15	269	0.17	
**Polycystic ovary syndrome**							< 0.0001
No	1,489,528	99.29	1,332,154	99.33	157,374	98.94	
Yes	10,640	0.71	8948	0.67	1692	1.06	
**Preeclampsia or hypertension**							< 0.0001
No	1,427,271	95.14	1,279,883	95.44	147,388	92.66	
Yes	72,897	4.86	61,219	4.56	11,678	7.34	
**Dyslipidemia**							< 0.0001
No	1,381,579	92.09	1,240,853	92.52	140,726	88.47	
Yes	118,589	7.91	100,249	7.48	18,340	11.53	

*GDM* Gestational diabetes mellitus.

^†^*p*-values from the Chi-square test.

Over a median follow-up of 12.8 years, there were 13,222 incident CVD cases (including 785 MIs, 298 coronary revascularization, 2705 heart failure, and 10,015 cerebrovascular disease), yielding an incidence rate of 68.95 per 100,000 person-years. The incidence rate of total CVD per 100,000 person-years was 76.78 among women with GDM compared with 68.06 in the reference group of parous women without GDM. Table 2 shows the association between GDM and incident CVD events. In the 4-year landmark analysis, GDM was a significant risk factor for subsequent CVD after controlling for age. Those with GDM experienced approximately 15% greater risk of CVD during follow-up (HR 1.15; 95% CI 1.09–1.22). The positive relationship between GDM and risk of CVD was attenuated but remained present after further adjustment for total parity, household income and history of preeclampsia or hypertension, polycystic ovary syndrome, and dyslipidemia (multivariable adjusted HR 1.08; 95% CI 1.02–1.14). As compared with women without GDM, age-adjusted HRs were significantly elevated in GDM group for each CVD subtype, except for myocardial infarction. The strongest associations with GDM were seen for incident coronary revascularization, followed by heart failure, and cerebrovascular disease. After adjusting for potential confounders, the presence of GDM was independently associated with a 58% (multivariable adjusted HR 1.58, 95% CI 1.16–2.14) and 20% (multivariable adjusted HR 1.20, 95% CI 1.07–1.35) increased risk of incident coronary revascularization and heart failure, respectively. Yet the positive associations of myocardial infarction and cerebrovascular disease with GMD were found to be non-significant.

**Table 2:** Results of landmark analysis for the association between gestational diabetes mellitus and risk of CVD.

Outcomes	No GDM (N = 1,341,102)			GDM (N = 159,066)			Age adjusted HR (95% CI)	P-value	Multivariable HR (95% CI)^b^	P-value
	No. of events†	Person-years	Incidence rate^a^	No. of events†	Person-years	Incidence rate^a^				
Total CVD	11,705	17,199,273	68.06	1517	1,975,840	76.78	1.15 (1.09–1.22)	< 0.0001	1.08 (1.02–1.14)	0.008
Myocardial infarction	693	17,233,934	4.02	92	1,980,253	4.65	1.20 (0.96–1.49)	0.107	1.12 (0.90–1.40)	0.314
Coronary revascularization	246	17,235,559	1.43	52	1,980,345	2.63	1.80 (1.34–2.44)	0.001	1.58 (1.16–2.14)	0.004
Heart failure	2367	17,230,905	13.74	338	1,979,655	17.07	1.32 (1.18–1.48)	< 0.0001	1.20 (1.07–1.35)	0.002
Cerebrovascular disease	8890	17,206,520	51.67	1125	1,976,892	56.91	1.11 (1.05–1.19)	0.001	1.04 (0.98–1.11)	0.184

*CVD* cardiovascular disease, *HR* hazard ratio, *CI* confidence interval.

^**†**^Note that the number of events in CVD subtypes does not add up to the total CVD cases as the subtypes are not mutually exclusive.

^a^Incidence rate per 100,000 person-years.

^b^Adjusted for age, parity, household income, history of preeclampsia or hypertension, polycystic ovary syndrome, and dyslipidemia.

Table 3 presents the hazard ratios for incident CVD associated with GDM, accounting for the intercurrent occurrence of T2DM. Characteristics of participants stratified into four groups (No GDM or T2DM, GDM only, T2DM only, GDM with progression to T2DM) are shown in Supplementary Table 1. Relative to the reference group of parous women with neither GDM nor T2DM, the corresponding age adjusted HRs for total CVD were 1.12 (95% CI 1.06–1.18) for women with GDM only, 3.09 (95% CI 2.51–3.82) and 3.64 (95% CI 3.03–4.38) for GDM with progression to T2DM and T2DM only, respectively. After further adjustment for relevant covariates, the HRs for total CVD were attenuated (GDM with progression to T2DM: HR 1.74, 95% CI 1.40–2.15; T2DM only group: HR 2.01, 95% CI 1.66–2.43), and there was no longer a statistically significant relationship between incident total CVD and GDM only (multivariable adjusted HR 1.06; 95% CI 1.00–1.12). Analyses of CVD subtypes indicated that the same is true of myocardial infarction and cerebrovascular disease. For example, GDM with subsequent progression to T2DM were linked with over two-fold increased risk of myocardial infarction and 1.55 times greater risk of cerebrovascular disease. However, for women with GDM in whom T2DM did not develop, the HRs for myocardial infarction and cerebrovascular disease were 1.08 (95% CI 0.86–1.37) and 1.03 (95% CI 0.97–1.10), respectively. On the contrary, GDM was significantly and positively associated with coronary revascularization and heart failure, regardless of intercurrent development of T2DM.

**Table 3:** Risk of incident CVD associated with GDM and progression to type 2 diabetes.

Categories	Events (N)	Incidence rate^a^	Age adjusted HR (95% CI)	Multivariable HR (95% CI)^b^
**Total CVD**				
No GDM or T2DM	11,590	67.54	1.00	1.00
GDM only	1428	73.67	1.12 (1.06–1.18)	1.06 (1.00–1.12)
T2DM only	115	295.09	3.64 (3.03–4.38)	2.01 (1.66–2.43)
GDM with progression to T2DM	89	237.18	3.09 (2.51–3.82)	1.74 (1.40–2.15)
**Myocardial infarction**				
No GDM or T2DM	686	3.99	1.00	1.00
GDM only	85	4.38	1.14 (0.91–1.43)	1.08 (0.86–1.37)
T2DM only	7	17.80	3.73 (1.77–7.86)	1.79 (0.79–4.05)
GDM with progression to T2DM	7	18.52	4.14 (1.96–8.73)	2.29 (1.07–4.88)
**Coronary revascularization**				
No GDM or T2DM	232	1.35	1.00	1.00
GDM only	44	2.27	1.66 (1.20–2.29)	1.53 (1.10–2.12)
T2DM only	14	35.61	19.11 (11.08–32.96)	8.97 (5.03–16.00)
GDM with progression to T2DM	8	21.16	11.98 (5.90–24.32)	5.51 (2.64–11.51)
**Heart failure**				
No GDM or T2DM	2333	13.57	1.00	1.00
GDM only	310	15.96	1.25 (1.11–1.41)	1.16 (1.03–1.31)
T2DM only	34	86.56	5.31 (3.78–7.46)	2.61 (1.84–3.71)
GDM with progression to T2DM	28	74.17	4.97 (3.42–7.22)	2.48 (1.70–3.63)
**Cerebrovascular disease**				
No GDM or T2DM	8810	51.32	1.00	1.00
GDM only	1065	54.92	1.09 (1.02–1.16)	1.03 (0.97–1.10)
T2DM only	80	204.71	3.29 (2.64–4.09)	1.85 (1.46–2.33)
GDM with progression to T2DM	60	159.63	2.69 (2.09–3.48)	1.55 (1.20–2.02)

*CVD* cardiovascular disease, *HR* hazard ratio, *CI* confidence interval, *GDM* gestational diabetes mellitus, *T2DM* type 2 diabetes mellitus.

^a^Incidence rate per 100,000 person-years.

^b^Adjusted for age, parity, household income, history of preeclampsia or hypertension, polycystic ovary syndrome, and dyslipidemia.

The post hoc power calculation indicated that the study had adequate statistical power to detect a clinically significant HR. With our sample size of 1,500,158, we had 78% power with a 5% significance level to detect a HR of 1.08 for total CVD outcome for GDM group vs non-GDM group^12,13^.

**Figure 1:**
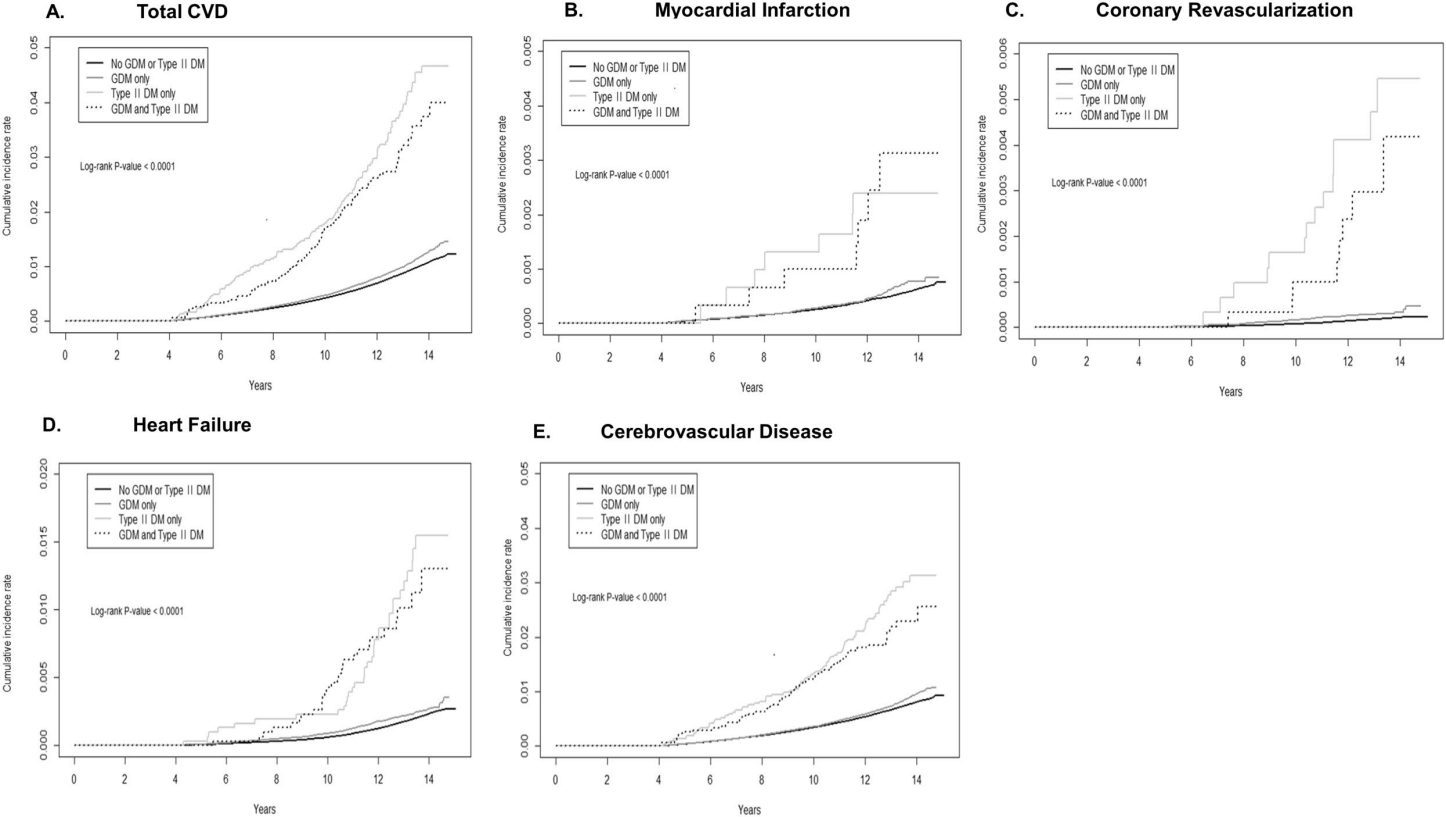
Kaplan–Meier curves for cumulative incidence of cardiovascular disease by GDM and T2DM status.

Figure 1 shows the Kaplan–Meier plot for the cumulative proportion of patients experiencing total CVD, including results of the log-rank test to compare incidence of CVD events between the exposure groups. The log-rank test revealed a statistically significant difference in the incidence of total CVD over time between the four exposure groups (P < 0.0001).

### Sensitivity analyses

In order to determine the sensitivity of the findings to the choice of landmark, we conducted a sensitivity analysis using an alternative landmark point at 2 years. As shown in Supplementary Table 2, the results of the sensitivity analysis were not materially different from the results of the main analysis, with a multivariable adjusted HRs for total CVD of 2.24 (95% CI 1.73–2.92), 2.09 (95% CI 1.59–2.75), and 1.11 (95% CI 1.04–1.18) in GDM with progression to T2DM, T2DM only, and GDM only groups, respectively. We also performed a sensitivity analysis of the primary analysis whereby all-cause deaths are included as endpoint events along with CVD. Participants who were still alive or without CVD by the end of the follow-up were treated as censored. There were no meaningful changes in the respective hazard ratios for the GDM with progression to T2DM (HR 1.66; 95% CI 1.37–2.01), T2DM only (HR 1.97; 95% CI 1.66–2.33), and GDM only groups (HR 1.04; 95% CI 0.99–1.09), highlighting the robustness of the primary results (Supplementary Table 3).

## Discussion

This large population-based retrospective study of Korean women demonstrated increased risk of cardiovascular events among individuals diagnosed with glucose intolerance during pregnancy. When examining CVD subtypes, we found that GDM is independently associated with a 58% and 20% increased risk of incident coronary revascularization and heart failure, respectively. More importantly, further analysis revealed that subsequent development of T2DM accounts for much of this elevated risk, which reinforces the importance of diabetes prevention strategies in this high-risk population.

Our estimates of the associations between GDM and future CVD are generally in accordance with those of previous studies. Based on a retrospective cohort study, Shah et al.^6^ found that the risk of incident CVD was significantly higher in women with previous GDM relative to age-matched controls. In their study, a hazard ratio of 1.71 (95% CI 1.08–2.69) was noted for CVD among women with GDM. Similar to our findings, the hazard ratio declined to 1.13 (95% CI 0.67–1.89) and lost its significance after further controlling for subsequent T2DM. Furthermore, in a meta-analysis of data pooled from 9 studies involving over five million participants, there was nearly twofold excess risk of cardiovascular events in women with history of GDM compared with non-GDM women (95% CI 1.57–2.50)^14^.

Several factors may underlie the observed relationship between GDM and cardiovascular outcomes. Among other potential mechanisms, GDM has shown to be related to diastolic dysfunction and a higher risk of adverse cardiac remodeling patterns, all of which are key factors involved in the development of heart failure^15,16^. Moreover, women with glucose intolerance during pregnancy were observed to exhibit higher left ventricular mass and wall thickness than people who are normoglycemic^17^, possibly explained by the enhanced formation of advanced glycation end products in the myocardium, which may result in endothelium damage and impaired arterial elasticity^18^. Previous research has reported significant abnormalities of cardiovascular system among 31 women with GDM compared with 34 healthy individuals^19^. There is also evidence that GDM is related to chronic insulin resistance, which could subsequently lead to metabolic disturbances^20^. In addition, variations in plasma cytokine levels and oxidative stress biomarkers have been documented among women with a history of GDM^21–23^. Di Cianni et al.^22^ observed that the prevalence of metabolic syndrome and c-reactive protein levels at 16 months after delivery are significantly higher in individuals with a previous history of GDM; inflammatory markers have been shown to predict future cardiovascular events^24^.

Our study findings have important clinical implications for the prevention of CVD among women with prior GDM. The public health burden of T2DM attributable to GDM is significant, with up to 31% of parous women who are diagnosed with T2DM having a history of GDM^25^. Moreover, a substantial proportion of women with previous GDM progress into abnormal glucose tolerance and T2DM^1,26,27^. The American Diabetes Association (ADA) guideline recommends that women with prior GDM or overt T2DM during pregnancy should undergo a 75 g oral glucose tolerance test (OGTT) for diabetes during 6 to 12 weeks postpartum and at least every 3 years thereafter^28^. Nevertheless, adherence to the recommendation is very low^29–32^. It is required to develop strategies to increase the rates of follow-up testing among the high-risk population. Moreover, lifestyle interventions targeting nutrition and physical activity have been shown to be effective in mitigating the progression to T2DM in individuals at risk for the disease, including women with a known history of GDM^33^. Therefore, in the future, a large well-designed clinical trials or community-based intervention studies are needed to ascertain the benefits of lifestyle or pharmacological interventions in reducing subsequent CVD risks among women with prior GDM.

There are several strengths to our analysis that add to the robustness of our study findings. These include the large sample size and the relatively long duration of follow-up, which allowed a high statistical power to study different types of CVD simultaneously. Furthermore, we adopted a landmark analysis to minimize the influence of guarantee-time bias. The current study also has some noteworthy limitations. Even though our findings were robust and consistent across a range of analyses, we cannot exclude the possibility that the observed associations have been subject to residual confounding, given the observational nature of the present study. In particular, our study used administrative data where information on clinical risk factors and other metabolic risk factors, such as gestational weight gain was not available^34^. We were therefore unable to delineate to what extent maternal conditions before or during pregnancy have affected the observed association between GDM and CVD. In addition, death from cardiovascular causes is an important composite outcome of cardiovascular diseases. Lack of such data in the NHIS claims data precluded its inclusion in the present study. Importantly, the claims data do not cover anthropometric and laboratory parameters, such as the oral glucose tolerance test, which is specific to GDM. This made it impossible to determine the severity of GDM. Further study is warranted to evaluate the impacts of severity of GDM on the risk of CVD.

In conclusion, this study provides evidence that women with a history of GDM are at increased risk of CVD, and subsequent progression to T2DM accounts for much of this elevated risk. These findings reinforce the need for implementation of postpartum diabetes interventions to reduce future CVD risk in women with a history of GDM.

## Methods

### Data source

This is a retrospective population-based study. The data used came from the 2002–2018 reimbursement claims database of Korea’s National Health Insurance Service (NHIS). The data has been described in detail elsewhere^35^. In short, as a single payer, the NHIS provides universal healthcare coverage for all residents in South Korea. The medical providers make claims to the Health Insurance Review and Assessment (HIRA) for reimbursement of the services they provide to patients. This process results in the compilation of a comprehensive source of healthcare utilization data, including demographics, the International Classification of Diseases 10th revision (ICD-10) principal diagnosis codes, procedure codes, prescription records, type of insurance, and medical care costs. This study was approved by the Institutional Review Board (IRB) of Severance Hospital, Yonsei University College of Medicine, Seoul, Korea (IRB no: Y-2018-0121) and the study complied with the Declaration of Helsinki for medical research involving human subjects. The requirement for informed consent was waived as the NHIS database was constructed after anonymization according to strict confidentiality guidelines.

### Study population

**Figure 2:**
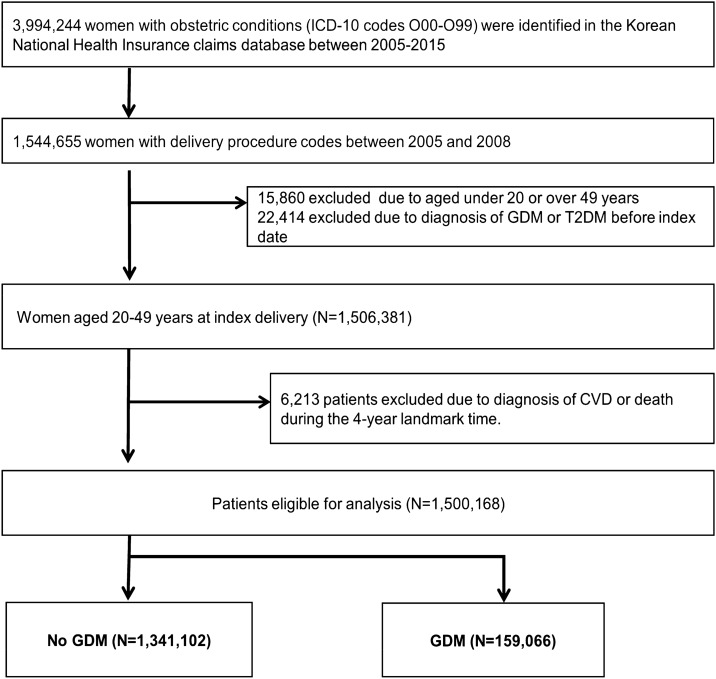
Flow chart of participant selection.

From the NHIS claims database, we identified a total of 3,994,244 women with obstetric conditions between 1 January 2005 and 31 December 2015 using the International Classification of Disease 10th revision [ICD-10] codes O00-O99. Among them, 1,544,655 women had given birth between January 1, 2005 and December 31, 2008. Procedure codes were used to identify vaginal and cesarean deliveries and the date of the first claim indicating delivery was used as the index delivery. For each participant, index date (date of conception) was calculated by subtracting 266 days (38 weeks) from the date of index delivery^36,37^. Our analysis was restricted to women aged between 20 to 49 years. To ensure that incidence risk, based on the period from the first GDM or T2DM occurrence to cardiovascular disease (CVD), could be accurately estimated, prevalent cases with GDM and T2DM before the index were excluded from our study. Additionally, participants with death, or CVD diagnosis within 4 years from the index date were also excluded. Consequently, the final study cohort consisted of 1,500,168 women. The enrollment flowchart for this study is illustrated in Fig. 2.

### Exposure assessment

In Korea, all pregnant women between 24 and 28 weeks of pregnancy are screened for GDM. The one-step method or the two-step method are both used for diagnosing GDM^38^. In the two-step method, pregnancy women then undergo a 100-g, 3-h oral glucose tolerance test (OGTT) after the non-fasting 50-g challenge test. The threshold values based on the criteria of Carpenter and Coustan with two abnormal values required for diagnosing GDM^39^. Since data on the OGTT test are not available in the NHIS claims data, International Classification of Diseases (ICD) 10th revision codes O24.4 and O24.9 during pregnancy were used to identify GDM cases. We classified patients as having GDM if they were diagnosed by the predetermined time point (within 4 years from the index date) and as unexposed otherwise, regardless of subsequent changes in exposure status. In further analyses, we accounted for intercurrent T2DM by categorizing the exposure status into 4 groups: without GDM or T2DM, GDM only, T2DM only, or both GDM and T2DM. Subjects without GDM or T2DM were set as the reference group. Patients were classified as having T2DM if they were screened negative during pregnancy followed by at least one claim with a diagnosis of T2DM (ICD-10: E11), either in outpatient or inpatient care, and were prescribed at least one of the following antidiabetic drug: dipeptidyl peptidase-4 inhibitors, α‐glucosidase inhibitors, biguanides, meglitinides, sulfonylureas, thiazolidinedione, and insulin.

### Ascertainment of CVD outcomes

The primary outcome of interest was the first occurrence of any CVD, defined as hospitalization with a primary diagnosis of myocardial infarction (ICD-10: I21-23), treatment with coronary revascularization (Procedure code: M6651-2, M6561, M6563-4, M6571-2, O1641-2, O1647, OA641-2, OA647), heart failure (ICD-10: I50), and cerebrovascular disease (ICD-10: I60-69).

### Covariates

Potential confounders were selected based on a priori assumptions of their relationships with both GDM and CVD^40–42^. Confounders included maternal age at index date, parity number, household income, polycystic ovarian syndrome, history of preeclampsia or hypertension and history of dyslipidemia. Maternal age at index delivery was categorized as 20–24, 25–29, 30–34, 35–39, > 40 years. Total parity was assessed during the landmark time and was categorized as 1, 2, or ≥ 3. Average monthly insurance premium at year of the index date was used as proxy of household income and was categorized into four groups based on quartiles (Low, middle-low, middle-high, or high). Polycystic ovarian syndrome (PCOS) was identified based on the diagnosis of PCOS (ICD-10: E28.2). History of preeclampsia or hypertension and history of dyslipidemia was confirmed based on the presence of diagnostic codes (Preeclampsia or hypertension ICD-10: O13-16, I10-15, dyslipidemia ICD-10: E78).

### Statistical analysis

The data were presented as frequencies and percentages. The differences in patient characteristics between groups of GDM and non-GDM were compared using the Chi-square test. Incidence rates were expressed as the number of new cases of CVD per 100,000 person-years of follow-up. In this study, Cox proportional hazard models were used to assess the association between GDM and incident CVD events, using landmark analysis at 4-year time point. Specifically, in order to ensure that every participant had equal exposure window for GDM exposure and to avoid guarantee-time bias, we selected a fixed time point, known as the landmark time, and survival analysis was conducted on only those subjects who have remained event-free at the specified time period (4 years from the index date)^43,44^. If guarantee-time bias is not accounted for in the analysis, biased estimates of the exposure effects can be caused, in favor of the exposure group^45,46^. All participants were followed up from the landmark point until the outcome of interest, death, or the end of study (31 December, 2018), whichever occurred first. Women without the event of interest by the end of the study were censored. In addition, we used an exposure window of 2 years in a sensitivity analysis to evaluate whether the arbitrarily chosen landmark time would affect the study results. Furthermore, cumulative distribution functions for CVD were estimated using the Kaplan–Meier method and the log-rank test was used to compare incidence of CVD events between the exposure groups. Two-sided P values less than 0.05 were considered statistically significant. Statistical analyses were performed using the SAS ver. 9.4 software and R 3.5.2 (R Foundation for Statistical Computing, Vienna, Austria). Post-hoc power estimation was performed using PASS 2019 software. SAS and all other SAS Institute Inc. product or service names are registered trademarks or trademarks of SAS Institute Inc. in the USA and other countries.

## Supplementary Information

Supplementary Information.

## Data Availability

All data generated or analysed during this study are not publicly available due to restrictions by the Korean NHIS. However, interested parties may submit applications to NHIS for access (http://nhiss.nhis.or.kr).
