# Supplementary material for: Gestational diabetes mellitus and the role of intercurrent type 2 diabetes on long-term risk of cardiovascular events
Source: Sci Rep. 2021 Oct 27;11:21140. doi: 10.1038/s41598-021-99993-4 (PMC8551203; doi:10.1038/s41598-021-99993-4)
Supplement: Supplementary file 1 — Supplementary Information. [file 41598_2021_99993_MOESM1_ESM.docx]

**Gestational Diabetes Mellitus and the Role of Intercurrent Type 2 Diabetes on Long-term risk of Cardiovascular Events**

Jiyu Sun*^1^, PhD, Gyu Ri Kim*^2,3^, PhD,

Su Jin Lee^,4^, MD, PhD, Hyeon Chang Kim^2^, MD, PhD

^1^Medical Research Collaborating Center, SMG-SNU Boramae Medical Center, Seoul, Korea

^2^Department of Preventive Medicine, Yonsei University College of Medicine, Seoul, Korea.

^3^Institute of Health Services Research, Yonsei University, Seoul, Korea

^4^Department of Internal Medicine, Seoul Red Cross Hospital, Seoul, Korea

*JS and GRK contributed equally to this work

**Co-corresponding authors:**

**Su Jin Lee**

Department of Internal Medicine, Seoul Red Cross Hospital, Seoul, Korea

**Hyeon Chang Kim**

Department of Preventive Medicine, Yonsei University College of Medicine, Seoul, Korea.

**Email addresses:**

JS jiyu.sun0@gmail.com

GRK [gyurikim@yuhs.ac](mailto:gyurikim@yuhs.ac)

SJL kibalhae82@gmail.com

HCK hckim@yuhs.ac

SUPPLEMENTARY TABLE

Sun J, Kim GR, et al. **Gestational Diabetes Mellitus and the Role of Intercurrent Type 2 Diabetes on Long-term risk of Cardiovascular Events.** *Scientific Reports*

**Supplementary Table 1.** Participant characteristics at index date according to GDM and intercurrent T2DM

**Supplementary Table 2.** Results of the sensitivity analyses using alternative landmark point at 2 years

**Supplementary Table 3 .** Association of GDM and progression to type 2 diabetes with risk of incident CVD and all-cause mortality

**Supplementary Table 4.** General characteristics of the study population in a subset of participants with body mass index, smoking status and drinking frequency measured

**Supplementary Table 5.** Risk of incident CVD associated with GDM and progression to type 2 diabetes controlling for body mass index, smoking status, drinking frequency and other covariates.

SUPPLEMENTARY TABLE

**Supplementary Table 1. Participant characteristics at index date according to GDM and intercurrent T2DM**

|  |  |  |  | **GDM status** | | | | | | |  |  |
| --- | --- | --- | --- | --- | --- | --- | --- | --- | --- | --- | --- | --- |
|  | **Total** | |  | **No GDM or T2DM** | | **GDM only** | | **T2DM only** | | **GDM with progression to T2DM** | | **P-value** |
|  | **N** | **%** |  | **N** | **%** | **N** |  | **N** | **%** | **N** | **%** |  |
| **Number** | 1,500,168 | 100.00 |  | 1,338,040 | 89.20 | 156,043 | 10.40 | 3062 | 0.20 | 3023 | 0.20 |  |
| **Age at index delivery, years** |  |  |  |  |  |  |  |  |  |  |  | <0.0001 |
| 20-24 | 119,566 | 7.97 |  | 111,377 | 8.32 | 7,884 | 5.05 | 182 | 5.94 | 123 | 4.07 |  |
| 25-29 | 624,771 | 41.65 |  | 559,431 | 41.81 | 63,517 | 40.70 | 935 | 30.54 | 888 | 29.37 |  |
| 30-34 | 589,440 | 39.29 |  | 522,181 | 39.03 | 64,846 | 41.56 | 1166 | 38.08 | 1247 | 41.25 |  |
| 35-39 | 149,382 | 9.96 |  | 130,454 | 9.75 | 17,658 | 11.32 | 628 | 20.51 | 642 | 21.24 |  |
| ≥40 | 17,009 | 1.13 |  | 14,597 | 1.09 | 2,138 | 1.37 | 151 | 4.93 | 123 | 4.07 |  |
| **Socioeconomic status** |  |  |  |  |  |  |  |  |  |  |  |  |
| \| Low income \| 335,050 \| 22.84 \|  \| 298814 \| 22.85 \| 34681 \| 22.66 \| 776 \| 26.28 \| 779 \| 26.42 \|  \| \| --- \| --- \| --- \| --- \| --- \| --- \| --- \| --- \| --- \| --- \| --- \| --- \| --- \| | 335,050 | 22.84 |  | 298,814 | 22.85 | 34,681 | 22.66 | 776 | 26.28 | 779 | 26.42 |  |
| Middle-low | 423,376 | 28.86 |  | 378,186 | 28.92 | 43,449 | 28.39 | 917 | 31.05 | 824 | 27.95 |  |
| Middle-high | 471,121 | 32.13 |  | 419,946 | 32.11 | 49,423 | 32.29 | 870 | 29.46 | 882 | 29.92 |  |
| High income | 237,242 | 16.17 |  | 210,901 | 16.13 | 25,488 | 16.65 | 390 | 13.21 | 463 | 15.71 |  |
| **Total parity** |  |  |  |  |  |  |  |  |  |  |  | <0.0001 |
| 1 | 1,283,451 | 85.55 |  | 1.144.617 | 85.55 | 133,379 | 85.48 | 2778 | 90.73 | 2677 | 88.55 |  |
| 2 | 214,411 | 14.29 |  | 191.394 | 14.30 | 22,399 | 14.35 | 276 | 9.01 | 342 | 11.31 |  |
| ≥3 | 2,306 | 0.15 |  | 2.029 | 0.15 | 265 | 0.17 | 8 | 0.26 | 4 | 0.13 |  |
| **Polycystic ovary syndrome** |  |  |  |  |  |  |  |  |  |  |  | <0.0001 |
| No | 1,489,528 | 99.29 |  | 1,329,276 | 99.35 | 154,423 | 98.96 | 2,878 | 93,99 | 2,951 | 97.62 |  |
| Yes | 10,640 | 0.71 |  | 8,764 | 0.65 | 1,620 | 1.04 | 184 | 6.01 | 72 | 2.38 |  |
| **Preeclampsia or hypertension** |  |  |  |  |  |  |  |  |  |  |  | <0.0001 |
| No | 1,427,271 | 95.14 |  | 1,277,764 | 95.50 | 145,247 | 93.08 | 2,119 | 69.20 | 2,141 | 70.82 |  |
| Yes | 72,897 | 4.86 |  | 60,276 | 4.50 | 10,796 | 6.92 | 943 | 30.80 | 882 | 29.18 |  |
| **Dyslipidemia** |  |  |  |  |  |  |  |  |  |  |  | <0.0001 |
| No | 1,381,579 | 92.09 |  | 1,239,426 | 92.63 | 139,460 | 89.37 | 1,427 | 46.60 | 1,266 | 41.88 |  |
| Yes | 118,589 | 7.91 |  | 98,614 | 7.37 | 16,583 | 10.63 | 1,635 | 53.40 | 1,757 | 58.12 |  |
| T2DM: type 2 diabetes mellitus; GDM: gestational diabetes mellitus; N: Number | | | | | |  |  |  |  |  |  |  |

**Supplementary Table 2. Results of the sensitivity analyses using alternative landmark point at 2 years**

|  | **Hazard ratio (95% CI)** | | | | | | | | |  |
| --- | --- | --- | --- | --- | --- | --- | --- | --- | --- | --- |
|  | **No GDM or T2DM (N=1,393,506)** |  | **GDM only (N=105,901)** |  | **T2DM only (N=1,449)** | |  | | **GDM with progression to T2DM (N=1,504)** | |
| **Total CVD** |  |  |  |  |  | |  | |  | |
| Age adjusted | 1.00 |  | 1.18 (1.11 to 1.25) |  | 3.31 (2.54 to 4.31) | |  | | 3.60 (2.79 to 4.66) | |
| Multivariable adjusted^a^ | 1.00 |  | 1.11 (1.04 to 1.18) |  | 2.09 (1.59 to 2.75) | |  | | 2.24 (1.73 to 2.92) | |
| **Myocardial infarction** |  |  |  |  |  | |  | |  | |
| Age adjusted | 1.00 |  | 1.31 (1.06 to 1.64) |  | 2.74 (0.88 to 8.52) | |  | | 5.53 (2.47 to 12.35) | |
| Multivariable adjusted^a^ | 1.00 |  | 1.23 (0.98 to 1.54) |  | 1.76 (0.56 to 5.52) | |  | | 3.34 (1.48 to 7.58) | |
| **Coronary revascularization** |  |  |  |  |  | |  | |  | |
| Age adjusted | 1.00 |  | 1.71 (1.23 to 2.41) |  | 12.53 (5.15 to 30.46) | |  | | 17.28 (8.13 to 36.74) | |
| Multivariable adjusted^a^ | 1.00 |  | 1.49 (1.05 to 2.10) |  | 5.82 (2.34 to 14.49) | |  | | 7.68 (3.49 to 16.86) | |
| **Heart failure** |  |  |  |  |  | |  | |  | |
| Age adjusted | 1.00 |  | 1.28 (1.12 to 1.45) |  | 5.11 (3.17 to 8.23) | |  | | 5.31 (3.29 to 8.56) | |
| Multivariable adjusted^a^ | 1.00 |  | 1.19 (1.05 to 1.36) |  | 3.17 (1.95 to 5.14) | |  | | 3.13 (1.93 to 5.09) | |
| **Cerebrovascular disease** |  |  |  |  |  | |  | |  | |
| Age adjusted | 1.00 |  | 1.15 (1.07 to 1.23) |  | 3.05 (2.23 to 4.18) | |  | | 3.16 (2.31 to 4.31) | |
| Multivariable adjusted^a^ | 1.00 |  | 1.08 (1.01 to 1.16) |  | 1.93 (1.39 to 2.68) | |  | | 1.96 (1.42 to 2.71) | |
| CVD: cardiovascular disease; GDM, gestational diabetes mellitus; T2DM: type 2 diabetes mellitus, CI: confidence interval | | | | | |  | |  | |  |
| ^b^Adjusted for age, parity, household income, history of preeclampsia or hypertension, polycystic ovary syndrome, and dyslipidemia | | | | | | | | | |  |

| **Supplementary Table 3. Association of GDM and progression to type 2 diabetes with risk of incident CVD and all-cause mortality** | | | | | |
| --- | --- | --- | --- | --- | --- |
| **Categories** | **Events (N)** | **Incidence rate^a^** | **Age adjusted HR (95% CI)** |  | **Multivariable HR (95% CI)^b^** |
| **Total CVD and all-cause mortality** |  |  |  |  |  |
| No GDM or T2DM | 16,585 | 96.65 | 1.00 |  | 1.00 |
| GDM only | 2,005 | 103.44 | 1.09 (1.05 to 1.15) |  | 1.04 (0.99 to 1.09) |
| T2DM only | 146 | 374.63 | 3.29 (2.79 to 3.89) |  | 1.97 (1.66 to 2.33) |
| GDM with progression to T2DM | 113 | 301.14 | 2.78 (2.31 to 3.34) |  | 1.66 (1.37 to 2.01) |
| **All-cause mortality** |  |  |  |  |  |
| No GDM or T2DM | 5655 | 32.88 | 1.00 |  | 1.00 |
| GDM only | 650 | 33.46 | 1.04 (0.96 to 1.12) |  | 0.99 (0.91 to 1.08) |
| T2DM only | 43 | 109.26 | 2.87 (2.12 to 3.87) |  | 2.05 (1.50 to 2.80) |
| GDM with progression to T2DM | 26 | 68.75 | 1.88 (1.28 to 2.77) |  | 1.27 (0.85 to 1.91) |
| ^a^Incidence rate per 100,000 person-years |  |  |  |  |  |
| ^b^Adjusted for age, parity, household income, history of preeclampsia or hypertension, polycystic ovary syndrome, and dyslipidemia | | | | | |
|  | | | | | |

**Supplementary Table 4 . General characteristics of the study population in a subset of participants with body mass index, smoking status and drinking frequency measured**

|  | **Total** | |
| --- | --- | --- |
|  | **N** | **%** |
| **Number** | 514,473 | 100.00 |
| **Age at index delivery, years** |  |  |
| 20-24 | 26,833 | 5.22 |
| 25-29 | 258,589 | 50.26 |
| 30-34 | 191,524 | 37.23 |
| 35-39 | 32,823 | 6.38 |
| ≥40 | 4,704 | 0.91 |
| **Socioeconomic status** |  |  |
| Low income | 112,366 | 21.84 |
| Middle-low | 167,875 | 32.63 |
| Middle-high | 176,546 | 34.32 |
| High income | 51,156 | 9.94 |
| Missing | 6,530 | 1.27 |
| **Total parity** |  |  |
| 1 | 435,841 | 84.71 |
| 2 | 78,038 | 15.17 |
| ≥3 | 594 | 0.12 |
| **Polycystic ovary** **syndrome** |  |  |
| No | 510,098 | 99.15 |
| Yes | 4,375 | 0.85 |
| **Preeclampsia or hypertension** |  |  |
| No | 491,201 | 95.48 |
| Yes | 23,272 | 4.52 |
| **Dyslipidemia** |  |  |
| No | 475,918 | 92.51 |
| Yes | 38,555 | 7.49 |
| **Body mass index** |  |  |
| Underweight | 80,085 | 15.57 |
| Normal | 334,081 | 64.94 |
| Overweight | 57,306 | 11.14 |
| Obese | 42,771 | 8.31 |
| Missing | 230 | 0.04 |
| **Drinking frequency** |  |  |
| Non-drinker | 306,770 | 59.63 |
| 2–3 times a month | 132,393 | 25.73 |
| 1–2 times a week | 58,777 | 11.43 |
| 3–4 times a week | 6,013 | 1.17 |
| Almost everyday | 926 | 0.18 |
| Missing | 9,594 | 1.86 |
| **Smoking status** |  |  |
| Non-smokers | 473,084 | 91.96 |
| Past smokers | 12,281 | 2.39 |
| Current smokers | 12,019 | 2.33 |
| Missing | 17,089 | 3.32 |

**Supplementary Table 5. Risk of incident CVD associated with GDM and progression to type 2 diabetes controlling for body mass index, smoking status, drinking frequency and other covariates**

| **Categories** | **N** | **Cases** |  | **Multivariable HR (95% CI)^a^** |  |
| --- | --- | --- | --- | --- | --- |
| **Total CVD** |  |  |  |  |  |
| No GDM or T2DM | 449,648 | 2,990 |  | 1.00 |  |
| GDM only | 63,329 | 429 |  | 1.05 (0.95 to 1.17) |  |
| T2DM only | 685 | 18 |  | 1.92 (1.20 to 3.07) |  |
| GDM with progression to T2DM | 811 | 21 |  | 1.92 (1.23 to 2.99) |  |
| **Myocardial infarction** |  |  |  |  |  |
| No GDM or T2DM | 449,648 | 158 |  | 1.00 |  |
| GDM only | 63,329 | 22 |  | 0.94 (0.59 to 1.50) |  |
| T2DM only | 685 | 3 |  | 4.73 (1.44 to 15.55) |  |
| GDM with progression to T2DM | 811 | 0 |  | - |  |
| **Coronary revascularization** |  |  |  |  |  |
| No GDM or T2DM | 449,648 | 54 |  | 1.00 |  |
| GDM only | 63,329 | 12 |  | 1.30 (0.67 to 2.50) |  |
| T2DM only | 685 | 3 |  | 6.52 (1.88 to 22.61) |  |
| GDM with progression to T2DM | 811 | 2 |  | 3.76 (0.85 to 16.59) |  |
| **Heart failure** |  |  |  |  |  |
| No GDM or T2DM | 449,648 | 534 |  | 1.00 |  |
| GDM only | 63,329 | 81 |  | 1.14 (0.90 to 1.45) |  |
| T2DM only | 685 | 9 |  | 4.31 (2.17 to 8.53) |  |
| GDM with progression to T2DM | 811 | 6 |  | 2.70 (1.18 to 6.17) |  |
| **Cerebrovascular disease** |  |  |  |  |  |
| No GDM or T2DM | 449.648 | 2,352 |  | 1.00 |  |
| GDM only | 63329 | 329 |  | 1.02 (0.90 to 1.15) |  |
| T2DM only | 685 | 8 |  | 1.14 (0.57 to 2.29) |  |
| GDM with progression to T2DM | 811 | 13 |  | 1.53 (0.86 to 2.72) |  |
| Abbreviations: CVD: cardiovascular disease; HR: hazard ratio; CI: confidence interval; GDM: gestational diabetes mellitus; T2DM: type 2 diabetes mellitus | | | | | |
| ^a^Adjusted for age, parity, household income, history of preeclampsia or hypertension, polycystic ovary syndrome, dyslipidemia, smoking status, drinking frequency and body mass index | | | | | |
|  | | | | | |
